# Current and Emerging Strategies for Myopia Control in Children: A Comprehensive Evidence-Based Review

**DOI:** 10.3390/jcm15041545

**Published:** 2026-02-15

**Authors:** Aldo Vagge, Matteo Baldi, Maria Musolino, Veronica Rivarone, Carlo Catti, Michele Iester

**Affiliations:** 1 University Eye Clinic of Genoa, Department of Neuroscience, Rehabilitation, Ophthalmology, Genetics, Maternal and Child Health (DiNOGMI), University of Genoa, Viale Benedetto XV, 5, 16132 Genoa, Italy; 4214937@studenti.unige.it (M.B.); maria.musolino@unige.it (M.M.); 4644389@studenti.unige.it (V.R.); 3660746@studenti.unige.it (C.C.); iester@unige.it (M.I.); 2 IRCCS “San Martino” Polyclinic Hospital, Largo Rosanna Benzi, 10, 16132 Genova, Italy

**Keywords:** myopia, myopia control, axial length, atropine, defocus spectacle lenses, contrast-modulating diffusion optics, orthokeratology, repeated low-level red-light therapy, combination therapy

## Abstract

Myopia has emerged as a global public health crisis, with prevalence exceeding 80% in East Asian urban populations and rising rapidly worldwide. High myopia substantially increases the lifetime risk of sight-threatening complications, including myopic macular degeneration, retinal detachment, and glaucoma. Multiple interventions have been investigated to slow myopia progression in children. Behavioral strategies, particularly increased outdoor exposure, demonstrate protective effects against myopia onset and may modestly slow progression, whereas several historically used approaches show no clinically meaningful benefit. Spectacle lens interventions include simultaneous defocus designs (e.g., DIMS, HALT, CARE) and contrast-modulating diffusion optics (DOT) lenses; collectively, these technologies have demonstrated consistent and clinically meaningful reductions in axial elongation across randomized clinical trials. Contact lens modalities, including dual-focus soft lenses and orthokeratology, have also demonstrated substantial efficacy in slowing progression in controlled studies. Low-dose atropine remains a cornerstone pharmacological therapy, particularly at concentrations between 0.01% and 0.05%, offering significant efficacy with minimal side effects. Repeated low-level red-light therapy has shown promising short-term reductions in axial elongation, although long-term safety and rebound effects remain uncertain. Combination therapy targeting complementary optical and pharmacological pathways shows additive benefits, particularly in children inadequately controlled with monotherapy. Contemporary clinical management emphasizes risk stratification based on axial length, age-specific growth targets, and structured longitudinal monitoring. The goal of modern myopia management is not merely to slow progression, but to prevent high myopia and reduce the lifetime burden of vision-threatening complications through a proactive, individualized approach increasingly regarded as the standard of care.

## 1. Introduction

Myopia, or nearsightedness, is a refractive condition characterized by the elongation of the eyeball, causing distant objects to appear blurred as light rays focus anterior to the retina. While traditionally considered a benign and easily correctable condition, myopia has now emerged as a major global public health concern due to its rapidly increasing prevalence and its association with sight-threatening complications in high and pathologic forms, including myopic maculopathy, retinal detachment, glaucoma, and choroidal neovascularization [1]. The World Health Organization and the International Myopia Institute have recognized myopia as a growing global public health concern [2,3]. Based on epidemiological modeling by Holden et al., it has been projected that nearly 50% of the global population may be myopic and approximately 10% may have high myopia by 2050, although recent analyses suggest that these projections may overestimate future prevalence due to limitations in data representativeness and modeling assumptions [4,5].

The epidemiological distribution of myopia is highly skewed by geography, urbanization, and lifestyle. The most alarming rates have been observed in East and Southeast Asia, where up to 80–90% of high school graduates are myopic and 10–20% progress to high myopia [6]. Countries such as China, South Korea, Japan, Singapore, and Taiwan have experienced a rapid rise, largely attributed to increased educational pressures, prolonged near-work, and limited time spent outdoors. Although the epidemic is most pronounced in Asia, similar upward trends have been reported in Western nations, with studies from the United States, Australia, and parts of Europe indicating a doubling of myopia prevalence in recent decades [4].

The clinical significance of myopia extends far beyond the need for optical correction. High myopia, defined as spherical equivalent ≤−6.00 D or axial length ≥26 mm, substantially increases the lifetime risk of vision-threatening pathologies [1,7]. Myopic maculopathy represents the leading cause of irreversible vision loss in highly myopic individuals, while the risk of retinal detachment increases approximately 20-fold compared to emmetropic eyes. Furthermore, myopia is associated with elevated risks of glaucoma and earlier onset of cataracts. Importantly, these risks are not binary but increase progressively with ocular growth. Axial length–based analyses show a strong dose–response relationship between increasing axial length and the risk of visual impairment and myopic maculopathy [8]. In parallel, population-based estimates indicate that each additional diopter of myopia is associated with an approximate 67% increase in the risk of myopic maculopathy, underscoring the clinical relevance of even modest reductions in myopia progression [9]. Eyes with axial lengths of 26–28 mm demonstrate an odds ratio of 3.07 for visual impairment by age 60 or older, while this risk escalates to 9.69–11.01 for axial lengths of 28–30 mm. Most strikingly, eyes with axial lengths ≥30 mm face an odds ratio of 24.69–93.62–up to 94-fold higher risk than eyes with normal axial length (<24 mm) [8]. This dose–response relationship underscores the importance of slowing myopia progression at any stage—every diopter of myopia prevented translates to meaningful reduction in lifetime ocular morbidity risk.

The economic burden of myopia is equally substantial, with global costs exceeding $200 billion annually when considering both direct healthcare costs and productivity losses [10].

Visual signals, particularly peripheral hyperopic defocus—where light focuses behind the peripheral retina—trigger compensatory mechanisms that stimulate ocular growth. This phenomenon is mediated by neuromodulators such as dopamine, nitric oxide, and retinoic acid, which influence biochemical pathways in the retina and sclera, altering scleral extracellular matrix remodeling and leading to thinning and elongation of the posterior globe [11]. Experimental evidence from animal models supports the theory that manipulating peripheral retinal image quality can directly influence axial length growth, forming the scientific basis for modern optical interventions [12].

## 2. Interventions Without Demonstrated Efficacy

Before discussing effective interventions, it is essential to acknowledge strategies that have been scientifically evaluated and found ineffective. According to the World Society of Pediatric Ophthalmology and Strabismus Myopia Consensus Statement 2025, numerous methods previously attempted by physicians and optometrists have yielded zero to statistically and clinically non-significant effects on slowing myopia progression [13]. The Consensus specifically requires that all interventions included in their recommendations demonstrate both statistical and clinical significance.

Among the interventions that have failed to demonstrate efficacy are under-correction of myopia, pinhole glasses, blue light blocking glasses, standard bifocal glasses, conventional progressive addition spectacle lenses, positively aspherized progressive addition lenses, daytime single vision soft contact lenses, and rigid gas permeable contact lenses for daytime wear [3,13]. These interventions have been subjected to rigorous evaluation through randomized controlled trials and systematic reviews, and the evidence base is now sufficiently robust to exclude these modalities from myopia management protocols.

Table 1 summarizes the interventions without demonstrated efficacy.

## 3. Behavioral and Environmental Interventions

Behavioral and environmental factors play a critical role in the development and progression of myopia, particularly in children. The evidence for the protective effect of increased time spent outdoors comes from multiple study types, including randomized controlled trials [14,15,16,17,18,19,20]. Multiple systematic reviews and meta-analyses have affirmed a 24–46% reduction in relative risk of incident myopia for every additional hour of outdoor time per week [21].

While it had been suggested that increased time spent outdoors was protective only in preventing myopia onset and not in slowing progression in already myopic eyes, six interventional studies and a meta-analysis have shown mitigation of myopia progression with a pooled reduction effect of 0.13 to 0.16 D per year [22]. Taking all the literature into account, approximately two hours of daylight exposure appears to mitigate the onset of myopia. It is luminance measured in lux that appears to be the critical factor: greater than 3000 lux appears protective, with levels >5000 lux showing stronger effects. Research has found that outdoor illumination can reach up to 15,000 lux on sunny days, while indoor light levels are typically much lower (often <100 lux). However, the specific protective threshold appears to be exposure to bright outdoor light (>3000 lux) for 40–120 min per day [23]. The mechanism underlying outdoor protection involves the release of retinal dopamine triggered by bright light exposure, a neuromodulator that inhibits ocular elongation [24].

Regarding near-work, a meta-analysis of studies across five continents found that children who engage in more near-work activities have an 80% higher risk of developing myopia, and each additional hour of near-work per week increases the odds by 2% [25]. However, the evidence is not entirely clear-cut, as several studies have not found this association when controlling for parental myopia and outdoor exposure. The intensity of near-work appears more important than frequency: continuous reading periods ≥30 min and close reading distance ≤30 cm were significantly associated with more myopia [26]. The COVID-19 pandemic provided natural experimental data, with studies showing a noticeable increase in myopia progression during lockdowns, particularly in younger children aged 6–8 years [27]. One study emphasized that the greatest increase was related to the period with most reduced outdoor time, further supporting the protective role of outdoor exposure [28]. Clinical recommendations therefore emphasize regular breaks during near-work, maintaining a reading distance of at least 30 cm, and adopting the “20–20–2 rule”: after 20 min of close work, children should gaze into the distance for at least 20 s and aim to accumulate approximately 2 h of outdoor time daily, as suggested in current clinical guidance [29].

## 4. Optical Treatment

Modern optical interventions for myopia control are designed to manipulate retinal defocus or contrast, which are key visual signals regulating eye growth and refractive development. The concept underlying most optical strategies is based on evidence that peripheral hyperopic defocus stimulates axial elongation, whereas peripheral myopic defocus inhibits it. Animal studies demonstrated that the peripheral refraction of the retina can influence ocular growth, with relative hyperopic defocus inducing myopia and myopic defocus inducing hyperopia [12]. Human studies showed that although relative peripheral hyperopic defocus cannot predict myopia development, those who became myopic developed relative peripheral hyperopia while those who remained emmetropic retained peripheral myopia [30]. Conventional single-vision spectacle lenses have been shown to increase peripheral hyperopic defocus in a dose-dependent fashion, providing no myopia control benefit [12,30].

It is critically important to recognize that not all defocus lens designs are equivalent in their myopia control efficacy. Spectacle lenses designed to reduce peripheral hyperopia without creating simultaneous myopic defocus—sometimes termed Peripheral Hyperopia Reduction Lenses—have shown minimal or no clinically significant effect on myopia progression [31]. These early designs, which aimed simply to neutralize peripheral hyperopic defocus, failed to provide the simultaneous competing myopic defocus signal that appears necessary for meaningful myopia control. In contrast, lenses incorporating simultaneous myopic defocus technology—where treatment zones create myopic defocus that competes with the corrected central image simultaneously across the retina—have demonstrated substantially greater efficacy, with treatment effects ranging from 40% to 67% reduction in progression [32]. This distinction underscores a fundamental principle: the retina requires a competing myopic signal, not merely the absence of hyperopic defocus, to effectively slow axial elongation. Current evidence supports simultaneous defocus designs (DIMS, HALT, CARE) as well as contrast-modulating diffusion optics (DOT) lenses over peripheral hyperopia reduction approaches.

The Defocus-Incorporated Multiple Segment (DIMS) spectacle lens consists of a central distance optical zone with diameter of 9 mm, surrounded by a honeycomb structure containing 396 small round segments approximately 1.03 mm in diameter with +3.50 diopters of defocus power, simultaneously allowing for clear central vision while introducing myopic defocus in the peripheral retina [33]. In a 2-year study involving 160 Chinese children, DIMS lenses significantly reduced myopia progression compared with single-vision lenses (0.41 vs. −0.85 D) and slowed axial elongation (0.21 vs. 0.55 mm). This corresponded to a 52% reduction in refractive progression and a 62% reduction in axial growth, with 21.5% of children wearing DIMS showing no progression versus 7.4% in the control group [33]. Subsequent studies showed that this effect was sustained at 3 years, with no evidence of rebound after stopping treatment [34]. Atropine drops showed an additive synergistic effect when used in conjunction with DIMS spectacles in children who continued to progress [35].

The HALT spectacle lenses incorporate Highly Aspherical Lenslet Target (HALT) technology, featuring 11 concentric rings containing 1021 aspherical lenslets that create a volume of non-focused light positioned in front of the retina [36]. In the pivotal study of 157 children aged 8–13 years, highly aspherical lenslets reduced myopia progression by 0.99 D and axial elongation by 0.41 mm over two years among full-time wearers compared with single-vision lenses, with ocular growth in approximately 90% of children similar to or slower than that of non-myopic peers [37,38]. Five-year follow-up data published in 2025 showed that children wearing HAL lenses experienced −1.27 D of myopia progression versus an estimated −3.03 D in extrapolated controls, with axial elongation of 0.67 ± 0.06 mm compared with 1.40 mm, corresponding to a between-group difference of 0.72 ± 0.10 mm [39].

An enhanced iteration of highly aspherical lenslet technology (HALT MAX) has been developed with increased lenslet power and asphericity to generate a broader volume of non-focused light positioned further in front of the retina (1.6 mm vs. 0.7 mm depth; 2.3 mm vs. 1.2 mm) [40]. In a randomized, contralateral, crossover study involving 50 Singaporean children aged 6–10 years, HALT MAX lenses demonstrated significantly slower axial elongation than standard HALT lenses across both study phases. The estimated cumulative one-year difference was 0.107 mm, with 78% and 72% of eyes wearing HALT MAX showing slower elongation in phases 1 and 2, respectively. Greater benefit was observed among children with faster baseline axial elongation, consistent with a potential dose–response relationship between lenslet optical characteristics and myopia control efficacy. Cumulative changes in non-cycloplegic spherical equivalent refraction at 12 months were approximately −0.21 D with HALT MAX compared with −0.42 D with standard HALT; however, these findings should be interpreted cautiously given the exploratory nature of the refractive analysis and absence of cycloplegia. While these results are promising, confirmation in larger, multicenter trials will be important to establish the generalizability of this approach [40].

CARE spectacle lenses incorporate cylindrical annular refractive elements (CARE) arranged as multiple micro-cylinders in concentric rings surrounding a central correction zone [41]. The design introduces relative positive power within the treatment zone; however, the precise mechanisms underlying myopia control remain incompletely understood. Differences in lens features, including central zone diameter and cylindrical element power, may influence treatment efficacy, although further investigation is needed to clarify these relationships [41]. The design philosophy aims to provide robust peripheral myopic defocus while maintaining excellent visual acuity and minimizing peripheral blur that could affect visual comfort. Compared with single-vision lenses, CARE spectacles slowed myopia progression by 0.44 D in spherical equivalent and reduced axial elongation by 0.20 mm over two years [41]. While the magnitude of effect appears somewhat lower than that reported in separate trials of certain lenslet-based designs, cross-study comparisons should be interpreted cautiously given differences in study populations and methodologies. CARE lenses therefore represent a validated addition to the myopia control armamentarium, particularly for patients who may prefer or require an alternative optical design [41].

Diffusion Optics Technology (DOT) spectacle lenses represent an alternative approach based on contrast modulation rather than traditional defocus, designed to slow myopia progression by slightly lowering retinal contrast and mimicking a more natural visual environment [42]. The contrast hypothesis proposes that abnormal contrast signaling between neighboring cones may stimulate axial elongation; however, the biological mechanisms underlying this process remain incompletely understood [43].

The Control of Myopia Using Peripheral Diffusion Lenses Efficacy and Safety Study (CYPRESS) was a multicenter, randomized, controlled, double-masked trial enrolling 256 children aged 6–10 years across 14 North American sites [42]. DOT lenses significantly slowed myopia progression, with sustained reductions in axial length and spherical equivalent reported through four years of follow-up [44]. Notably, CYPRESS is among the first spectacle lens trials to demonstrate efficacy in an ethnically diverse North American pediatric population, expanding the evidence base beyond predominantly Asian cohorts.

Recent developments in lens technology suggest that mechanisms beyond peripheral myopic defocus may contribute to myopia control. Lenslet-ARray-Integrated (LARI) lenses are available in two configurations featuring either positive (+3.00 D) or negative (−3.00 D) power lenslets, both designed to provide a clear central visual field while producing similar retinal image blur within the lenslet array zone [45]. A randomized, double-masked controlled trial involving 240 Chinese children demonstrated that both designs significantly slowed myopia progression compared with single-vision lenses (1-year progression: −0.30 D and −0.21 D vs. −0.66 D; axial elongation: 0.19 mm and 0.17 mm vs. 0.34 mm), with no significant differences between PLARI and NLARI [45].

These findings suggest that optical signals other than the sign of defocus may play a role in regulating eye growth; however, the underlying mechanisms remain speculative and warrant further investigation.

The peripheral defocus framework has historically guided optical myopia control, supported by animal and experimental evidence demonstrating that hyperopic defocus promotes axial elongation whereas myopic defocus inhibits eye growth [11]. However, emerging data suggest that additional optical mechanisms may influence ocular growth beyond defocus alone [45]. Lenslet-ARray-Integrated lenses provide a notable example: despite opposite lenslet powers, both configurations produce similar retinal image blur and demonstrate comparable efficacy in slowing myopia progression [45].

These findings support the hypothesis that reduced peripheral image contrast, or more broadly a controlled degradation of peripheral retinal image quality, may represent a shared downstream mechanism across effective optical interventions. This contrast-modulation effect has also been proposed for diffusion optics spectacle lenses and may contribute to the efficacy of simultaneous defocus designs and orthokeratology, all of which alter peripheral retinal image quality through different optical principles [3,42,43].

Importantly, this emerging interpretation should be viewed as a refinement and extension of the current defocus-based model rather than definitive evidence against it. Further confirmation from independent studies and diverse populations is required to clarify the relative contributions of defocus, contrast modulation, and higher-order aberrations in myopia control. The next challenge for research will be to elucidate the underlying neural mechanisms and to determine the optimal degree of peripheral image degradation necessary to achieve maximal treatment efficacy while maintaining acceptable visual quality.

Table 2 summarizes the efficacy of spectacle lens–based myopia control interventions, reporting both relative efficacy estimates and absolute changes in spherical equivalent refraction (diopters) and axial length (millimeters), derived from the primary results reported in the present Section and the cited trials, to facilitate clinical interpretation. This approach improves clinical interpretation and comparison across different optical strategies.

## 5. Contact Lens Interventions

The 2025 Cochrane living systematic review included 12 randomized controlled trials involving 1338 participants comparing multifocal soft contact lenses with single-vision lenses. The analysis demonstrated a consistent treatment effect across follow-up periods: at one year, the mean difference in spherical equivalent was 0.27 D (95% CI 0.18 to 0.35), and 0.30 D (95% CI 0.19 to 0.41) at two years. Axial elongation was reduced by −0.11 mm at one year (95% CI −0.13 to −0.09) and −0.15 mm at two years (95% CI −0.19 to −0.12) [46]. Evidence beyond two years remains limited, and uncertainty persists regarding cumulative effects [46].

### 5.1. Dual-Focus Soft Contact Lenses

Dual-focus soft contact lenses are daily disposable contact lenses composed of Omafilcon A material and feature a central correction zone of 3.36 mm surrounded by concentric rings alternating between distance and near powers, producing +2.00 D of simultaneous myopic retinal defocus [47]. A pivotal three-year randomized clinical trial enrolling 144 children demonstrated significantly slower myopia progression compared with single-vision lenses (−0.51 ± 0.64 D vs. −1.24 ± 0.61 D) and reduced axial elongation (0.30 ± 0.27 mm vs. 0.62 ± 0.30 mm), corresponding to approximately 59% and 52% reductions, respectively [48]. Long-term follow-up indicated a cumulative treatment effect over six years, with children initially assigned to treatment maintaining slower progression rates. Participants who crossed over from single-vision lenses also experienced substantial reductions in axial elongation after initiating therapy [49].

These lenses received FDA approval in 2019 for myopia control in children aged 8–12 years, representing the first contact lens specifically approved for this indication in the United States [50].

### 5.2. Other Multifocal and EDOF Designs

The BLINK study evaluated center-distance multifocal soft contact lenses with medium (+1.50 D) and high (+2.50 D) add powers versus single-vision lenses in 294 children [51]. Only the high-add group demonstrated clinically meaningful and statistically significant differences compared with controls, with a between-group difference of approximately 0.46 D in spherical equivalent and −0.23 mm in axial length over three years [51]. Extended depth of focus (EDOF) contact lenses represent an alternative optical approach. Sankaridurg et al. evaluated two EDOF contact lens designs against single-vision soft contact lenses (SVSCLs) and found significantly less myopia progression over two years, with reductions ranging from 0.23 to 0.31 D and axial elongation decreases of approximately 0.13–0.14 mm [52]. In contrast, Shen et al., using a contralateral design in which one eye wore an EDOF lens and the fellow eye an SVSCL, reported a modest reduction in refractive progression after one year (0.18 D) and a small between-eye difference in axial elongation (~0.04 mm), the clinical relevance of which remains uncertain [53]. Overall, EDOF lenses appear to provide a moderate benefit in slowing refractive progression, although evidence for axial length control remains less consistent [52,53]. Positive spherical aberration lenses demonstrated a mean difference of approximately 0.14 D in spherical equivalent and −0.14 mm in axial length versus control at one year [54].

### 5.3. Orthokeratology

Overnight orthokeratology uses reverse geometry rigid gas-permeable lenses worn during sleep to temporarily flatten the central cornea, providing correction-free daytime vision while inducing relative peripheral myopic shift. The 2025 Cochrane Review included 13 studies with 1373 participants, reporting reduced axial elongation across both study durations: −0.18 mm at one year (95% CI −0.21 to −0.14) and −0.30 mm at two years (95% CI −0.38 to −0.23) [51]. Evidence from multiple orthokeratology studies, including randomized and prospective trials such as ROMIO and TO-SEE, indicates that treatment slows axial elongation compared with conventional correction. Across studies, axial length increased by approximately 0.3 mm in children wearing orthokeratology lenses versus about 0.6 mm in control groups over two years, corresponding to an average reduction of roughly 50%. The treatment effect appears to be greater in younger children and is most pronounced during the first one to two years of therapy, although continued—albeit smaller—benefits may persist thereafter [55]. Combining orthokeratology with low-dose atropine (0.01%) demonstrated an additive effect, slowing axial elongation by approximately 28% compared with orthokeratology alone over two years [56].

Potential complications of orthokeratology include microbial keratitis, with an initial US study estimating risk in children of 13.9 per 10,000 patient-years, compared to 7.7 per 10,000 in all wearers, although this estimate was associated with wide confidence intervals [57]. More recent evidence from a large pediatric cohort in Russia suggests a lower incidence of approximately 5 per 10,000 patient-years (95% CI 2.1–12.4), comparable to daily wear soft contact lenses, possibly reflecting improvements in standardized fitting protocols and patient education [58].

Table 3 summarizes contact lens interventions for myopia control.

## 6. Pharmacological Treatment with Atropine

The use of atropine for myopia control is not a recent discovery. As early as 1916, Pollock in Glasgow reported treating myopic children with atropine for six years, observing that suspension of accommodation over considerable periods was beneficial, in some cases arresting or even reducing myopia [59]. Despite nearly a century of clinical use and robust evidence from 26 randomized controlled trials included in the 2025 Cochrane Review, the exact mechanism of action remains incompletely understood—a paradox that continues to intrigue researchers [60].

Atropine is a non-selective muscarinic antagonist, and while it causes cycloplegia, evidence strongly supports a non-accommodative mechanism for its anti-myopia effect. Key evidence includes the observation that atropine prevents myopia in chicks, whose ciliary muscle is innervated by nicotinic rather than muscarinic receptors; bifocals and progressive lenses that reduce accommodative demand fail to control myopia; and the retina and sclera, rather than the ciliary muscle, appear to be the primary sites of action [61]. However, the muscarinic receptor hypothesis has been challenged by observations that atropine inhibits glycosaminoglycan synthesis in isolated scleral cells without acetylcholine present, and that many other muscarinic antagonists fail to prevent myopia despite similar receptor binding profiles [62]. The most plausible current hypothesis involves retinal signaling pathways that modulate scleral extracellular matrix remodeling, though critical questions regarding specific receptor subtypes and tissue barriers remain unresolved.

In 2006, the Atropine for the Treatment of Myopia One (ATOM 1) study showed that nightly 1% atropine reduced myopia progression by approximately 77% over two years, with minimal axial elongation compared with a mean increase of about 0.38 mm in controls [63]. In 2012, the subsequent ATOM2 trial demonstrated that 0.01% atropine retained clinically meaningful efficacy while producing minimal side effects, including an average pupil dilation of about 1 mm, a modest reduction in accommodation, and no significant impairment of near visual acuity [64].

The Low-Concentration Atropine for Myopia Progression (LAMP) study provided critical dose-finding data. In Hong Kong children aged 4–12 years randomized to 0.05%, 0.025%, 0.01%, or placebo, spherical equivalent progression was reduced by 67%, 43%, and 27% respectively, and axial length growth was slowed by 51%, 29%, and 12% after one year [65]. The LAMP phase 2 trial showed that 0.05% atropine remained the most effective concentration among those tested, with approximately double the efficacy of 0.01% over two years [66]. Over five years, continued 0.05% atropine treatment has shown good efficacy with cumulative mean spherical equivalent progressions of −1.34 D, −1.97 D, and −2.34 D for the 0.05%, 0.025%, and 0.01% groups, respectively. Most children needed to restart treatment after cessation and restarting with 0.05% achieved similar efficacy as continued treatment [67].

A 2022 network meta-analysis involving 30 pairwise comparisons from 16 randomized controlled trials with 3272 participants ranked 1%, 0.5%, and 0.05% atropine as the three most beneficial concentrations for myopia control. When assessed by relative risk for overall myopia progression, 0.05% atropine was ranked as the most beneficial (RR 0.39; 95% CI 0.27–0.57) [68]. Geographic and ethnic variability in response to low-dose atropine has been increasingly recognized. The MOSAIC randomized clinical trial conducted in Ireland demonstrated significant reductions in spherical equivalent refraction and axial elongation at 24 months (difference 0.13 D and 0.09 mm, respectively), although treatment effects varied depending on the analytical approach [69]. Similarly, the Western Australia ATOM study reported a mild-to-moderate treatment benefit at 18 months that was no longer statistically significant after two years [70]. In a US randomized clinical trial, low-dose 0.01% atropine showed limited efficacy in slowing myopia progression, whereas the large multicenter CHAMP trial across North America and Europe has shown reductions in both refractive progression and axial elongation compared with placebo, despite complexities related to endpoint hierarchy [71,72]. Differences in treatment response may reflect ethnic and biological factors, as efficacy was influenced by iris color in the MOSAIC trial, with greater treatment effects observed in lighter-colored eyes [69].

The CHAMP trial reported that 0.01% atropine slowed axial elongation by 0.13 mm compared with placebo, whereas the higher 0.02% concentration produced a smaller reduction of 0.08 mm, suggesting a response that was not strictly dose-dependent [72]. In the LAMP randomized trial, 0.05% atropine was the most effective concentration for reducing both spherical equivalent progression and axial elongation over one year, although this greater efficacy was accompanied by larger changes in pupil diameter and accommodation [65].

A landmark development occurred in June 2025 when the European Commission approved preservative-free atropine 0.01%, a 0.01% atropine formulation, making it the first pharmaceutical agent approved specifically for myopia control in the European Union [73]. The indication is for children aged 3–14 years with myopia between −0.50 D and −6.00 D showing progression of ≥0.50 D per year. Approval was based on the phase 3 STAR trial involving 847 children, demonstrating a 30% reduction in annual myopia progression over two years compared with placebo [74]. Japan had previously approved a 0.025% atropine formulation in December 2024, representing the first ophthalmic solution authorized in the country to slow myopia progression [75]. The US FDA subsequently issued a Complete Response Letter acknowledging that the primary endpoint had been met but concluding that the available data did not support the effectiveness of low-dose atropine for pediatric myopia, although no safety or product-quality concerns were identified [76].

Adverse effects appear concentration-dependent. In ATOM1, withdrawal due to allergic reactions, glare, and blurred near vision occurred in 4.5%, 1.5%, and 1% of participants receiving 1% atropine, with no serious treatment-related adverse events reported [63]. ATOM2 demonstrated improved tolerability with 0.01% atropine compared with higher concentrations, alongside sustained efficacy over five years; a dose-related rebound was observed after treatment cessation, yet 0.01% remained the most effective regimen overall [64,77]. Long-term observational follow-up from the Atropine Treatment Long-term Assessment Study (ATLAS) reported no ocular complications attributable to atropine exposure over 10–20 years [78]. Consistently, a network meta-analysis confirmed a dose-dependent increase in adverse effects, with lower concentrations showing more favorable profiles for pupil size and accommodation [68].

Overall, atropine appears to represent a cornerstone pharmacological intervention in contemporary myopia management, with efficacy strongly influenced by concentration, age, population characteristics, and treatment duration [51,65,68]. Among low-dose formulations, 0.05% atropine demonstrates favorable efficacy in Asian populations, while response to lower concentrations appears more variable and may depend on individual patient characteristics, ethnicity, and baseline progression rates [67]. Lower concentrations (0.01–0.025%) may be particularly appropriate in older children, slower progressors, or as an initial conservative approach with favorable safety profile [65,66,67,68].

Table 4 summarizes atropine efficacy by concentration.

## 7. Repeated Low-Level Red-Light Therapy

Repeated low-level red-light (RLRL) therapy represents an emerging intervention using low-intensity 650–660 nm red light, typically administered for 3 min twice daily, 5 days per week [79]. The proposed mechanism involves photobiomodulation enhancing mitochondrial activity, ATP production, and choroidal blood flow, counteracting the choroidal thinning associated with myopia progression [79]. The 2025 Cochrane Review network meta-analysis found that repeated low-level red-light (RLRL) therapy significantly slowed axial elongation at one year, with a mean difference of −0.35 mm (95% CI −0.41 to −0.29) compared with control, although the certainty of the evidence was rated as very low. The same analysis reported a reduction in myopia progression of approximately 0.84 D (95% CI 0.63 to 1.05), again supported by very low-certainty evidence [46].

In a multicenter randomized trial enrolling 264 Chinese children aged 8–13 years, repeated low-level red-light (RLRL) therapy reduced spherical equivalent progression by 76.6% over 12 months compared with single-vision spectacles, and efficacy increased with higher compliance; choroidal thickness increased by 16.1 µm at 1 month [80]. In a post hoc analysis of the same randomized trial, axial length shortening >0.05 mm at 12 months was observed in 21.85% (26/119) of children receiving RLRL therapy versus 1.38% (2/145) of control [81].

However, concerns have been raised regarding a potential rebound effect following treatment cessation. A 2-year post-trial follow-up study reported a modest rebound after discontinuation of repeated low-level red-light therapy, with significantly greater axial elongation (0.42 mm vs. 0.28 mm) and myopic progression (−0.91 D vs. −0.54 D) observed in children who stopped treatment compared with controls [79]. These findings highlight ongoing uncertainty about the optimal duration of therapy and suggest that maintenance treatment strategies may need to be considered.

Repeated low-level red-light (RLRL) therapy appears generally well tolerated in the short term; a multicenter randomized clinical trial reported no functional vision loss or structural retinal changes on optical coherence tomography over 12 months, with only two participants discontinuing treatment because the light was perceived as too strong [80].

However, current evidence remains limited. A recent Cochrane systematic review concluded that available studies provide only preliminary evidence of efficacy and emphasized the need for longer-term investigations in more diverse populations to better define safety outcomes [3].

Additional expert analyses have suggested that some red-light devices may approach or exceed recommended exposure limits, raising a theoretical risk of photochemical or thermal retinal injury when treatment parameters are not adequately controlled, although causality has not been established [82].

Consistent with this uncertainty, RLRL therapy is not currently approved by major regulatory agencies, and all published clinical studies to date have been conducted in Asian populations, underscoring the need for further research before routine clinical adoption can be recommended [3].

## 8. Combination Therapy

Combining optical and pharmacological treatments targets different pathways: atropine affects biochemical and cellular scleral mechanisms while optical devices provide defocus signals. This dual-target strategy may provide additive or synergistic effects, particularly for children progressing despite monotherapy [35,83]. Since optical and pharmacological interventions likely work through distinct mechanisms, their combined use may yield benefits exceeding either approach alone.

Evidence for orthokeratology combined with low-dose atropine is emerging. A prospective randomized clinical trial reported that axial elongation over one year was significantly lower with orthokeratology plus atropine 0.01% (0.09 ± 0.12 mm) compared with orthokeratology alone (0.19 ± 0.15 mm), corresponding to a 53% greater effect in slowing axial growth [84]. Evidence from a recent Cochrane network meta-analysis indicates that combining orthokeratology with low-dose atropine is associated with an additional reduction in axial elongation of approximately 0.12 mm at one year compared with orthokeratology alone. Three studies directly comparing atropine 0.01% with orthokeratology found no clinically important difference in axial length after one year [46].

Detailed analyses indicate that the additional benefit of combination therapy remains stable over time: a randomized double-masked cross-over trial reported a mean reduction in axial elongation of 0.10 mm in the first year and 0.09 mm in the second year compared with orthokeratology alone [85]. Similarly, a randomized trial by Kinoshita et al. found 2-year axial length progression of 0.29 ± 0.20 mm with combination therapy versus 0.40 ± 0.23 mm with orthokeratology alone [56]. Combination therapy may therefore be considered in children with rapid axial elongation or poor response to orthokeratology alone [85].

Evidence for combining myopia control spectacle lenses with atropine is emerging. In a retrospective study of 107 Chinese children aged 7–12 years, Huang et al. reported that defocus-incorporated multiple segment (DIMS) lenses combined with 0.01% atropine resulted in myopia progression of 0.49 ± 0.66 D and axial elongation of 0.28 ± 0.24 mm over 12 months, compared with 0.79 ± 0.47 D and 0.41 ± 0.22 mm with DIMS alone, and 1.07 ± 0.64 D and 0.52 ± 0.22 mm in single-vision controls [35]. Nucci et al., in a prospective controlled observational study of 146 European children, reported that both DIMS spectacle lenses and 0.01% atropine significantly reduced myopia progression and axial elongation compared with single-vision controls, with the slowest progression observed in the combined atropine+DIMS group [86].

A particularly relevant clinical scenario involves children who continue to progress despite low-dose atropine monotherapy. Sim et al. conducted a prospective cohort study of 50 children (mean age 8.9 ± 1.1 years) who demonstrated ≥0.5 D progression over 6 months despite atropine 0.01% or 0.025%. Prior to combination therapy, myopia progressed by −0.60 ± 0.38 D with axial elongation of 0.24 ± 0.10 mm. After the addition of highly aspherical lenslet target (HALT) spectacle lenses while maintaining atropine, progression slowed significantly to −0.06 ± 0.38 D and 0.06 ± 0.10 mm at 6 months, and to −0.07 ± 0.28 D and 0.13 ± 0.14 mm over 12 months. A hyperopic axial shift was observed in 24% of children, and no complaints of glare, near blur, or peripheral blur were reported [87].

In 2024, Vagge et al. reported real-world data from a retrospective cohort of 175 Italian children comparing HALT spectacle lenses alone (n = 41), 0.01% atropine alone (n = 48), combination therapy (n = 33), and single-vision controls (n = 53). At 12 months, all treatment groups demonstrated significantly less myopia progression than controls. The combined therapy achieved greater control of spherical equivalent refraction than either monotherapy, while axial elongation was significantly lower than with atropine alone but not significantly different from HALT spectacle lenses [88].

## 9. Clinical Approach and Treatment Strategy

Modern myopia management increasingly relies on systematic and individualized approaches. The following section presents a proposal of an evidence-informed clinical framework intended to illustrate how the data reviewed in the preceding sections may be translated into practical clinical decision-making. This framework is not proposed as prescriptive guidance, but rather as an adaptable, illustrative approach grounded in published evidence, axial length–based risk stratification, and widely accepted clinical principles, and should be applied with appropriate clinical judgment.

### 9.1. Step 1: Spherical Equivalent and Axial Length Percentile Assessment

Clinical management begins with comprehensive assessment including history of onset age, parental myopia, outdoor time, and near-work habits; cycloplegic refraction using tropicamide 1% or cyclopentolate 1% administered as two drops five minutes apart; axial length by optical biometry (mean of three measurements); fundus examination for myopic changes; and binocular vision assessment [89,90].

Axial length percentiles, referenced to age and sex-matched normative data, provide one of the most objective metrics for identifying at-risk children and guiding treatment decisions. The use of gender-specific growth curves is essential, as axial length development differs between boys and girls [91].

If axial length exceeds the 50th percentile for age and sex, treatment should be considered. For children with axial length at or below the 50th percentile, close monitoring is warranted to detect percentile drift over time. A jump of more than 10–15 percentiles between visits indicates accelerated growth and should prompt treatment initiation regardless of absolute axial length value [91].

Percentile thresholds should be interpreted in the context of population-specific normative data and individual growth trajectories.

### 9.2. Step 2: Risk Factor Assessment

Consider additional risk factors that influence myopia progression and inform treatment intensity:

- Parental myopia: Presence of one or two myopic parents significantly increases progression risk. Children with one myopic parent have approximately twice the risk of developing myopia, while those with two myopic parents face a three- to five-fold increased risk. Children with strong familial predisposition are particularly susceptible to environmental drivers and may benefit from early implementation of lifestyle and therapeutic interventions [18,19].
- Environmental factors: Environmental factors significantly influence myopia development. Outdoor exposure of at least 2 h per day is associated with delayed myopia onset, though evidence for slowing progression remains limited. Prolonged near-work—particularly continuous periods exceeding 30 min and performed at working distances shorter than 30 cm—may further increase myopia risk. These modifiable factors should be systematically addressed through lifestyle counseling for all patients. A practical approach is the “20–20–2 rule”: after 20 min of close work, children should gaze into the distance for at least 20 s and aim to spend approximately 2 h outdoors each day, while maintaining a minimum working distance of 30 cm—although specific evidence supporting this combined protocol is limited [29].

### 9.3. Step 3: Treatment Selection

Treatment selection should consider patient age, axial length percentile, predicted treatment compliance (including child and family factors), and current scientific evidence. Each treatment modality has specific practical considerations [13].

Spectacle lenses offer a non-invasive approach suitable for all ages with no infection risk and no clinically significant rebound effect has been reported [3]. For children who are suitable candidates for contact lens wear, based on individual maturity, family support, hygiene, and clinician judgment, options include dual-focus soft contact lenses or orthokeratology [48,49,55]. While orthokeratology demonstrates excellent efficacy for myopia control, clinicians should carefully weigh its benefits against the potential risk of microbial keratitis, which, though rare, represents a serious sight-threatening complication. Rigorous patient selection, comprehensive hygiene education, and close monitoring are essential when considering this option [55].

For atropine, the 0.01 to 0.05% concentration is widely considered to provide a favorable balance of efficacy and tolerability [65,66,67,68,69,92].

Combination therapy, pairing optical interventions (spectacle lenses, soft contact lenses, or orthokeratology) with low-dose atropine, is typically considered for patients showing insufficient response to monotherapy. This approach should be considered at 6–12-month review if axial elongation exceeds age-matched physiological growth rate [3]. Emerging evidence suggests additive effects when combining these treatment modalities [13].

Management options for insufficient response may include increasing atropine concentration, adding atropine to optical intervention, or switching optical modality, while always verifying compliance [13].

Regardless of the treatment modality selected, essential lifestyle modifications should be recommended for all patients. These include at least 1–2 h of outdoor time daily in bright light and limiting continuous near-work sessions to 30 min or less while maintaining a reading distance of at least 20–30 cm [13].

### 9.4. Step 4: Follow-Up Strategy and Treatment Adjustment

Monitoring should occur usually every 6–12 months with axial length measurement using optical biometry and cycloplegic refraction as the primary outcome metric. Axial length should typically be monitored at approximately 6-month intervals, whereas cycloplegic refraction should be performed annually, or at an interval adjusted according to clinical indication [93].

Axial length, not refraction, represents the critical endpoint for myopia control and must be measured at each examination. Individual values should be plotted against age- and sex-matched normative data, taking into account population-specific growth patterns [29,94].

To facilitate the clinical application of these axial length growth curves in myopia management, a color-coded classification system based on annual axial elongation rates has been proposed [95]. The classification system employs three categories, each represented by a distinct color code for immediate visual recognition:

- Green zone (low/tolerable axial length growth): Indicates that axial elongation remains within physiological limits and that treatment may not be necessary.
- Yellow zone (moderate growth): Suggests an increased risk of progression and supports the initiation of myopia control therapy.
- Red zone (high growth): Reflects excessive elongation and warrants strong consideration of immediate and potentially intensified treatment.

### 9.5. Treatment Discontinuation

Treatment generally continues until age 15 years, when significant ocular growth is no longer expected. However, the specific timing of discontinuation depends primarily on the stabilization of axial length growth. Treatment discontinuation can be considered when axial length growth has stabilized, typically to less than approximately 0.1 mm per year for more than one year at age 15, or when the growth rate reaches 0.05 mm per year or less [29,91,94].

For optical interventions (spectacle lenses, multifocal contact lenses), cessation seems not to produce rebound effects, allowing for straightforward discontinuation once stabilization is achieved. However, orthokeratology discontinuation before age 14 has been associated with accelerated axial elongation, warranting continuation through adolescence [96].

Atropine cessation requires more careful management. Rebound effects, characterized by accelerated myopic progression following treatment discontinuation, are well-documented and influenced by multiple factors including age at cessation, treatment duration, baseline spherical equivalent, and atropine concentration. Children younger than 12 years, those with shorter treatment durations, and those using higher concentrations demonstrate greater rebound risk. For concentrations of 0.1% or higher, gradual tapering rather than abrupt cessation is generally recommended to minimize rebound. Lower concentrations and older age at cessation are associated with reduced rebound effects. Following atropine discontinuation, axial length should be monitored at 6-month intervals for at least 12 months to detect potential rebound, with readiness to reinitiate treatment if excessive progression resumes [96].

The decision to discontinue treatment should be individualized, considering the child’s age, progression history, final refractive error, and risk factors for continued progression. In some cases, particularly for children with persistent risk factors or family history of high myopia, extended treatment beyond traditional stopping points may be warranted [29,96].

Table 5 summarizes an evidence-informed clinical protocol for myopia management, integrating axial length–guided risk stratification with stepwise monitoring and treatment adjustment.

## 10. Conclusions

A proactive and individualized approach to myopia management—grounded in axial length–based risk stratification, evidence-based treatment selection, and objective longitudinal monitoring—is increasingly regarded as the standard of care. Importantly, even modest reductions in axial elongation during childhood can translate into meaningful decreases in the lifetime risk of sight-threatening myopic complications.

## Figures and Tables

**Table 1:** Interventions Without Demonstrated Efficacy for Myopia Control.

Intervention	Evidence
Under-correction of myopia	No benefit; may accelerate progression
Pinhole glasses	No effect on progression
Blue light blocking glasses	No effect on myopia progression
Standard bifocals/PALs	Minimal or no clinically significant effect
Peripheral Hyperopia Reduction Lenses	Minimal efficacy; simultaneous defocus required for meaningful effect
Daytime soft SVCLs	No myopia control benefit
Daytime RGP lenses	No myopia control benefit. May reduce spherical equivalent but show no effect on axial length elongation

**Table 2:** Spectacle Lens Interventions for Myopia Control.

Lens Technology	Mechanism	SER Change (D)	Axial Elongation (mm)	Efficacy (SER)	Efficacy (AL)
DIMS (MiYOSMART^®^)	396 segments, +3.50 D myopic defocus	−0.41 ± 0.06 vs. −0.85 ± 0.08 (2 y)	0.21 ± 0.02 vs. 0.55 ± 0.02 (2 y)	52%	62%
HALT (Stellest^®^)	1021 aspherical lenslets (VoNFL)	−0.99 D vs. SVL (2 y); −1.27 vs. −3.03 D (5 y)	0.41 mm vs. SVL (2 y); 0.67 ± 0.06 vs. 1.40 mm (5 y)	NR	0.41 mm/2 y; 0.72 ± 0.10 mm/5 y
HALT MAX (Stellest 2.0^®^)	Increased power and asphericity lenslets	−0.21 D vs. −0.42 D †	0.043 ± 0.016 vs. 0.105 ± 0.016 (phase 1); 0.077 ± 0.013 vs. 0.123 ± 0.014 (phase 2); cumulative difference 0.107 mm	NR	47% vs. HALT (1 y)
CARE (MyoCare^®^)	Cylindrical annular refractive elements	0.44 D reduction vs. SVL (2 y)	0.20 mm reduction vs. SVL (2 y)	37%	32.8%
DOT (SightGlass)	Contrast reduction via diffusers	0.33 D (3 y); 0.52 D (4 y)	0.13 mm (3 y); 0.20 ± 0.09 mm (4 y)	NR	0.32 mm/3 y
LARI (PLARI/NLARI)	Lenslet array with +3.00 D or −3.00 D lenslets producing comparable peripheral blur	−0.30/−0.21 vs. −0.66 D (1 y)	0.19/0.17 vs. 0.34 mm (1 y)	NR	NR

**Notes:** Cross-study comparisons should be interpreted cautiously due to differences in study design, sample characteristics, follow-up duration, and outcome definitions. SER and axial length values represent between-group differences versus single-vision spectacle lenses unless otherwise specified. NR = not reported in the referenced study. For HALT MAX, efficacy data are reported relative to the standard HALT configuration. † Measured by non-cycloplegic autorefraction. For LARI lenses, outcomes are reported separately for positive (PLARI) and negative (NLARI) configurations. **Abbreviations:** AL = axial length; CARE = Cylindrical Annular Refractive Elements; D = diopter(s); DIMS = Defocus Incorporated Multiple Segments; DOT = Diffusion Optics Technology; HALT = Highly Aspherical Lenslet Target; LARI = Lenslet Array Integrated; NLARI = Negative-power Lenslet Array Integrated; NR = not reported; PLARI = Positive-power Lenslet Array Integrated; SER = spherical equivalent refraction; SVL = single-vision lenses; VoNFL = Volume of Non-Focused Light; y = year / years.

**Table 3:** **Contact Lens Interventions for Myopia Control.**

Intervention	Mechanism	SER Change (D)	Axial Elongation (mm)
Dual-focus soft contact lenses	Peripheral myopic defocus	−0.51 ± 0.64 vs. −1.24 ± 0.61 (3 y)	0.30 ± 0.27 vs. 0.62 ± 0.30 (3 y)
Multifocal soft contact lenses	Peripheral myopic defocus	MD 0.27 (1 y); 0.30 (2 y); 0.47 (3 y)	−0.11 (1 y); −0.15 (2 y); −0.22 (3 y)
High-add center-distance multifocal lenses	Increased peripheral myopic defocus	−0.46 (3 y)	−0.23 (3 y)
EDOF lenses	Manipulation of retinal image quality/depth of focus	Reduction of 0.23–0.31 (2 y)	Reduction of 0.13–0.14 (2 y)
Positive spherical aberration lenses	Induced spherical aberration	0.14 vs. control (1 y)	0.14 vs. control (1 y)
Orthokeratology	Reverse geometry RGP lenses flatten central cornea, inducing relative peripheral myopic shift	Not primary outcome	−0.18 (1 y); −0.30 (2 y)

**Notes:** Cross-study comparisons should be interpreted cautiously due to heterogeneity in study populations, optical designs, add powers, outcome measures, and duration of follow-up. Absolute values represent mean differences versus single-vision soft contact lenses as reported in the original trials. Percent efficacy values are provided for contextual comparison. Where indicated, reductions refer to treatment effects relative to control groups. **Abbreviations**: AL = axial length; BLINK = Bifocal Lenses in Nearsighted Kids study; CI = confidence interval; D = diopter(s); EDOF = extended depth of focus; MD = mean difference; MFSCL = multifocal soft contact lens; NS = not statistically significant; SER = spherical equivalent refraction; SVL/SVSCL = single-vision lens/single-vision soft contact lens; y = year/years.

**Table 4:** Atropine Efficacy by Concentration.

Concentration	SER Change (D)	Axial Elongation (mm)	Relative Efficacy (AL)
1.0%	−0.28 ± 0.92	−0.02 ± 0.35	~100% (ATOM 1, 2 y) [Relative Efficacy (SER) 77%]
0.5%	−0.30 ± 0.60	0.27 ± 0.25	~64% (ATOM 2, 2 y)
0.05%	−0.55 ± 0.86	0.39 ± 0.35	~52% (LAMP, 2 y); ~55% (LAMP, 5 y)
0.025%	−0.85 ± 0.73	0.50 ± 0.33	~39% (LAMP, 2 y)
0.01%	−0.49 ± 0.63 (ATOM 2, 2 y); −1.12 ± 0.85 (LAMP, 2 y)	0.41 ± 0.32 (ATOM 2, 2 y); 0.59 ± 0.38 (LAMP, 2 y)	~46% (ATOM 2, 2 y); ~28% (LAMP, 2 y)

**Notes:** Absolute values represent mean changes in spherical equivalent refraction (SER) and axial length (AL) as reported in the original randomized clinical trials (primarily ATOM and LAMP). Relative efficacy values reflect the reported percentage reduction in myopia progression or axial elongation and are provided for contextual comparison across atropine concentrations. Because these values derive from different study populations, designs, and follow-up durations, they should not be interpreted as direct head-to-head comparisons. **Abbreviations:** SER = Spherical Equivalent Refraction; AL = Axial Length; D = Diopters; mm = millimeters; ATOM = Atropine for the Treatment of Myopia study; LAMP = Low-Concentration Atropine for Myopia Progression study; y = year/years.

**Table 5:** Evidence-informed clinical protocol for myopia management based on axial length–guided risk stratification.

	Clinical Domain	Key Assessment	Recommended Clinical Action
**1**	Baseline assessment	Family history Lifestyle factors Cycloplegic refraction Axial length Age- and sex-matched axial length percentile	Establish baseline risk profile
**2**	Risk stratification	Axial length percentile Cycloplegic spherical equivalent Parental myopia Outdoor exposure Near-work demand	Stratify patients
**3**	Treatment selection	Patient age Axial length percentile Growth velocity Expected compliance	Initiate evidence-based intervention adapted to local availability
**4**	Follow-up monitoring	Axial length (~6 months) Cycloplegic refraction (annually or on indication)	Assess treatment response and detect progression
	Treatment adjustment	Axial elongation exceeding physiological growth	Escalate or modify treatment strategy
**5**	Treatment discontinuation	Axial elongation stabilization through adolescence	Consider discontinuation with continued monitoring

## Data Availability

No new data were created or analyzed in this study.
